# Regulation of Human Sortilin Alternative Splicing by Glucagon-like Peptide-1 (GLP1) in Adipocytes

**DOI:** 10.3390/ijms241814324

**Published:** 2023-09-20

**Authors:** Ashley Lui, Rekha S. Patel, Meredith Krause-Hauch, Robert P. Sparks, Niketa A. Patel

**Affiliations:** 1 Department of Molecular Medicine, University of South Florida, Tampa, FL 33612, USA; ashleylui@usf.edu (A.L.); meredith.krause-hauch@va.gov (M.K.-H.); 2 Research Service, James A. Haley Veterans Hospital, Tampa, FL 33612, USA; rekha.patel1@va.gov (R.S.P.); robert.sparks@umassmed.edu (R.P.S.); 3 Department of Medicine, Division of Gastroenterology, UMass Chan Medical School, Worcester, MA 01655, USA

**Keywords:** type 2 diabetes, human adipocytes, human adipose tissue, glucose uptake, splicing minigene, antisense oligonucleotides, splice factors, TRA2B

## Abstract

Type 2 diabetes mellitus is a chronic metabolic disease with no cure. Adipose tissue is a major site of systemic insulin resistance. Sortilin is a central component of the glucose transporter -Glut4 storage vesicles (GSV) which translocate to the plasma membrane to uptake glucose from circulation. Here, using human adipocytes we demonstrate the presence of the alternatively spliced, truncated sortilin variant (Sort_T) whose expression is significantly increased in diabetic adipose tissue. Artificial-intelligence-based modeling, molecular dynamics, intrinsically disordered region analysis, and co-immunoprecipitation demonstrated association of Sort_T with Glut4 and decreased glucose uptake in adipocytes. The results show that glucagon-like peptide-1 (GLP1) hormone decreases Sort_T. We deciphered the molecular mechanism underlying GLP1 regulation of alternative splicing of human sortilin. Using splicing minigenes and RNA-immunoprecipitation assays, the results show that GLP1 regulates Sort_T alternative splicing via the splice factor, TRA2B. We demonstrate that targeted antisense oligonucleotide morpholinos reduces Sort_T levels and improves glucose uptake in diabetic adipocytes. Thus, we demonstrate that GLP1 regulates alternative splicing of sortilin in human diabetic adipocytes.

## 1. Introduction

Type 2 diabetes mellitus (T2DM) continues to be a rising epidemic with more than 34.2 million Americans diagnosed with T2DM in 2020 and 88 million living with prediabetes [1]. The prevalence of T2DM has steadily increased since the mid-1900s and is projected to reach 60.7 million Americans by 2060 [2]. T2DM, a chronic disease with no cure, is characterized by hyperglycemia and insulin resistance and promotes metabolic syndrome and comorbidities such as cardiovascular disease, neuropathy, and nephropathy. 

Adipose tissue is a major site of systemic insulin resistance contributing to T2DM progression and serves as a predictor for cardiovascular disease [3]. Nearly 90% of patients with T2DM are overweight or obese [1]. In adipocytes, the major glucose transporter, glucose transporter- 4 (Glut4), is tethered in the cytoplasm within Glut4 Storage Vesicles (GSVs) [4]. Postprandial signals translocate GSVs to the membrane, integrating Glut4 for glucose uptake from the circulation and GSVs are later recycled back through the trans-Golgi network [5]. Perturbed signaling in insulin-resistant adipocytes results in less translocation of GSVs to the plasma membrane and thus reduces glucose uptake, contributing to the diabetic pathology [6,7,8].

Components of GSVs have been identified [9,10,11,12] and of these, trafficking protein sortilin is an essential factor for GSV formation and function [13,14]. Sortilin is a multi-ligand sorting receptor ubiquitously expressed in the body. Its cellular role involves endocytosis of targets and trafficking of multiple vesicles through the trans-Golgi network as well as targeting to the lysosome for degradation. In adipocytes, sortilin’s luminal domain binds Glut4 [15], and its motif rich cytoplasmic tail guides sortilin through the trans-Golgi network via specific post translational modifications [16,17] and interactions with various proteins [18,19,20]. 

Alternative splicing increases protein diversity by generating distinct proteins from the same gene via inclusion and exclusion of exons or alternate 5′ or 3′ splice site usage. Alternative splicing produces multiple products, often with different functions, from the same gene. Mis-regulation of alternative splicing may contribute to or cause a disease [21]. An alternatively spliced human sortilin variant was identified in neurons with inclusion of an exon, named exon 17b, inserted between exons 17 and 18 in sortilin pre-mRNA [22,23]. Importantly, the mouse and human sortilin pre-mRNA sequences are different, and it has been demonstrated [22,23,24] that a longer sortilin splice variant is present in mouse cells, which is not seen in human cells. In neurons, sortilin functions as a receptor for progranulin, while in adipocytes, sortilin is a component of the GSVs and functions as a sorting protein. However, presence of alternatively spliced variants of sortilin in human adipocytes, and their roles in glucose metabolism are not yet reported. Since sortilin is a component of GSVs in adipocytes and glucose uptake is reduced in diabetes, we evaluated human adipose tissue and human adipocytes from normal and type 2 diabetic individuals for the presence of sortilin splice variants. Our results demonstrate presence of the truncated splice variant (Sort_T) in human adipose tissue and adipocytes whose expression is increased in T2DM. Based on this discovery, we evaluated the role of the splice variant Sort_T in glucose uptake in T2DM adipocytes. The results demonstrate that Sort_T over-expression decreases glucose uptake in adipocytes. Glucagon-like peptide-1 (GLP1), an incretin hormone, and its analogs regulate glucose uptake and are widely used as therapeutics to manage T2DM. However, the effect of GLP1 on the expression of sortilin splice variants is not yet known. Hence, we hypothesized that GLP1 may regulate the alternative splicing of sortilin in human adipocytes. We have systematically evaluated the underlying alternative splicing mechanisms of sortilin pre-mRNA regulated by GLP1 in diabetic adipocytes. 

## 2. Results

### 2.1. Alternatively Spliced Sortilin Variants in Human Adipocytes

**Figure 1:**
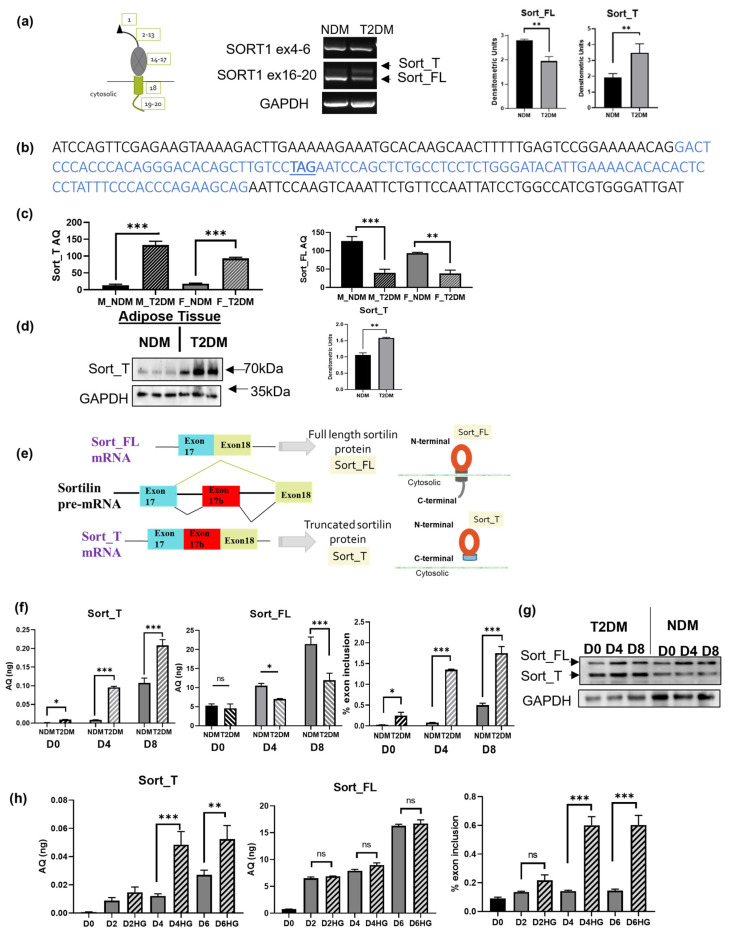
Sortilin alternatively spliced variants in diabetic human visceral adipocytes and adipose tissue. (**a**) HASCs from diabetic (DM) and nondiabetic (T2DM) donors were differentiated to mature adipocytes, RNA was isolated, and PCR performed using primers amplifying the region between exon 4 to 6 or the region between exon 16 to 20 (exon numbers of sortilin indicated in schematic). Products were separated on a 1% agarose gel and visualized using ethidium bromide staining. Graphs show densitometric analysis of individual PCR products normalized to GAPDH (n = 4). Statistical analysis was performed using *t*-test and ** *p* < 0.01. (**b**) The PCR products obtained using primers to sortilin cytoplasmic tail on exon 16 (sense) to exon 20 (antisense) were purified and sequenced. Sequencing of top band revealed inclusion of a 99bp sequence (as indicated by blue font), named exon 17b, between exons 17 and 18 of sortilin mRNA with an in-frame stop codon (bold, underlined). (**c**) Adipose tissue from male and female, nondiabetic and diabetic donors was homogenized, and RNA was isolated. Real-time QPCR was performed using primers specifically within exon 17b detecting Sort_T and primer on the exon 17 to exon 18 junction to detect Sort_FL. A standard curve for each sortilin variant was included with the qPCR assay along with samples analyzed in triplicate. Absolute quantification (AQ, ng) for Sort_FL and Sort_T was calculated by normalizing the values to GAPDH absolute levels. Statistical analysis was performed using *t*-test between T2DM and NDM within each gender, ** *p* < 0.01 and *** *p* < 0.001. (**d**) Adipose tissue from T2DM and NDM donors were homogenized and whole-cell lysates were harvested. Western blot was performed and immunoblotted using Sort_T specific antibody as indicated. Graphs show densitometric analysis of Sort_T normalized to GAPDH (n = 3). Statistical analysis was performed using two-tailed Student’s *t*-test and ** *p* < 0.01. (**e**) Schematic of sortilin pre-mRNA alternative splicing generating Sort_FL mRNA and Sort_T mRNA which produces Sort_FL protein (with C-terminal cytosolic tail) and Sort_T (truncated C-terminal). (**f**) T2DM and NDM hASCs were differentiated and harvested every four days (Day 0 preadipocytes: D0, Day 4 differentiated adipocytes: D4, Day 8 mature adipocytes: D8) to determine sortilin variant levels during adipogenesis. Real-time SYBR Green qPCR was performed using primers specifically for Sort_T and Sort_FL. A standard curve for each sortilin variant was included with the qPCR assay along with samples analyzed in triplicate. Graphs represent absolute quantification (AQ, ng) for Sort_FL and Sort_T calculated by normalizing to GAPDH AQ. Percent exon 17b inclusion was calculated using the following equation: $\%exon\;inclusion=\frac{Sort\_T}{Sort\_T+Sort\_FL}*100\%$. Statistical analysis was performed via multiple *t*-tests between diabetic and nondiabetic samples for each sortilin variant at different time points (n = 3). * *p* < 0.05, *** *p* < 0.001, ns = not significant. (**g**) Western blot was performed on NDM and T2DM D0, D4 and D8 whole-cell lysates using antibodies to N-terminal sortilin (detects both Sort_FL and Sort_T simultaneously) and GAPDH. Experiments repeated thrice. (**h**) NDM hASCs were differentiated in the presence of 25 mM glucose and harvested every 48 h along with a control sample. Real-time SYBR Green qPCR was performed. Graphs represent absolute quantification (AQ, ng) for Sort_FL and Sort_T calculated by normalizing to GAPDH and percent exon 17b inclusion was calculated using the following equation: $\%exon\;inclusion=\frac{Sort\_T}{Sort\_T+Sort\_FL}*100\%$. Statistical analysis was performed via multiple *t*-tests between control and high-glucose (HG) samples for each sortilin variant at different time points (n = 3). ** *p* < 0.01, *** *p* < 0.001, ns = not significant.

Human visceral adipose stem cells (hASCs; from ZenBio™, Durham, NC, USA) from pooled nondiabetic or type 2 diabetic donors were used for experiments. We have previously shown that these hASCs maintain the patient’s phenotype [25,26]. To evaluate the expression of sortilin, type 2 diabetic (T2DM) and nondiabetic (NDM) hASCs were differentiated to mature adipocytes, PCR was performed using primers targeting different domains of sortilin, and products were separated on a 1% agarose gel (Figure 1a). A single product was detected using primers that amplify sortilin’s exons 4 to 6 in both NDM and T2DM adipocytes. Two products were detected in T2DM adipocytes using primers that amplify sortilin’s exons 16–20. To determine the identity of these two PCR products, bands from the gel were purified and sequenced. Sequencing results (Figure 1b) indicated an inclusion of 99bp in the upper band, which corresponded to the exon 17b and confirmed the presence of the alternatively spliced variant as a result of exon 17b inclusion between exons 17 and 18 in human adipocytes. Further analysis of this sequence revealed an in-frame stop codon TAG (indicated by bold, underlined). Sequences were compared to exonic and intronic sequences from NCBI (Reference Sequence NM_002959.7, Gene ID: 6272 in *Homo sapiens*). The constitutively expressed product is referred to as Sort_FL, while the alternative variant is referred to as Sort_T. Our results demonstrate alternative splicing of sortilin in human adipocytes and increased levels of the alternatively spliced variant Sort_T in T2DM adipocytes.

### 2.2. Sort_T Is Increased in Diabetic Visceral Adipose Tissue

Human visceral adipose tissue from T2DM and NDM donors (de-identified, male and female, n = 8 each, NHSR determination) were obtained to determine the presence of sortilin variants. Real-time qPCR was performed using primers specifically on exon 17b detecting Sort_T and a primer on the exon 17 to exon 18 junction to detect Sort_FL. The results demonstrate a marked increase in Sort_T levels in T2DM adipose tissue with concurrent reduced Sort_FL levels compared to NDM adipose tissue. This pattern occurred in both male and female adipose tissue (Figure 1c). Interestingly, total sortilin levels are lower in females compared to males. To confirm protein levels of truncated sortilin variant in adipose tissue, a Western blot was performed (Figure 1d). Lysates from NDM and T2DM visceral adipose tissue were analyzed via Western blot using Sort_T specific antibody. The results show increased Sort_T protein expression in T2DM adipose tissue. Figure 1e shows a schematic of sortilin pre-mRNA alternative splicing generating Sort_FL mRNA and Sort_T mRNA, which produces Sort_FL protein (full length with C-terminal cytosolic tail) and Sort_T (truncated C-terminal, missing cytosolic tail), respectively. 

### 2.3. Differential Sortilin Splice Variant Expression in Nondiabetic and Diabetic Adipocytes during Adipogenesis

Next, we sought to elucidate the expression of sortilin splice variants during adipogenesis. The NDM and T2DM hASCs were differentiated and harvested as pre-adipocytes (D0), terminally differentiated adipocytes (D4), and mature adipocytes (D8). RNA was processed using real-time qPCR using primers specific for Sort_T and Sort_FL. The percent of Sort_T in total sortilin was calculated as percent exon inclusion for D0, D4 and D8 (Figure 1f). Western blot analysis was performed on whole-cell lysates and the results show high Sort_T expression levels during adipogenesis in T2DM compared to NDM (Figure 1g). These results demonstrate that Sort_T is significantly increased during adipogenesis in T2DM adipocytes.

### 2.4. Hyperglycemia Increases Expression of Sort_T during Adipogenesis

To elucidate the effect of hyperglycemia (hallmark of diabetes) on sortilin alternative splicing, NDM hASCs were grown and differentiated in the presence of 25 mM high glucose (HG) and collected every 48 h. Real-time qPCR was performed using primers specific for Sort_T and Sort_FL and results showed a marked increase in Sort_T levels and percent exon 17b inclusion in response to hyperglycemia (Figure 1h). 

### 2.5. Molecular Dynamics and Intrinsic Disorder Analysis of Sortilin Splice Variants

**Figure 2:**
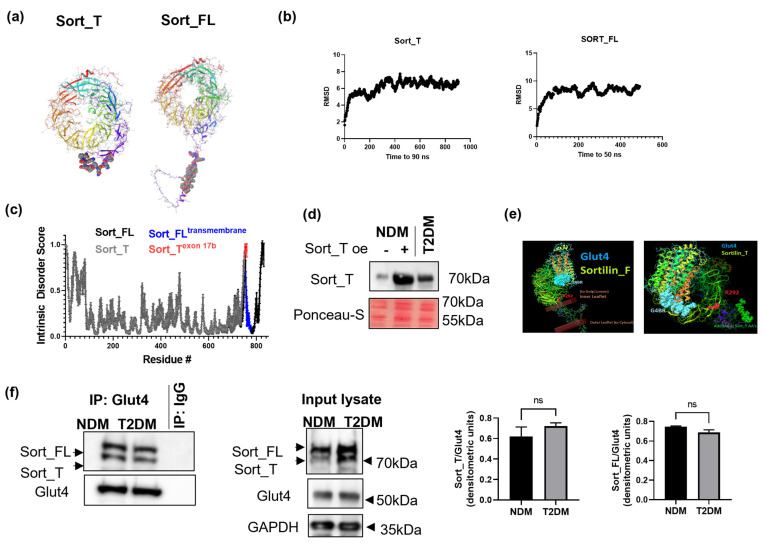
Molecular modeling and association of sortilin variants with Glut4. (**a**) De novo structure of sortilin splice variants was modeled using AlphaFold software. (**b**) Molecular dynamics (MD) was performed on the lowest energy structure for Sort_T and Sort_FL using Schrodinger software. RMSD was calculated, indicating stability over 100 ns MD minimization. (**c**) Intrinsic disorder was predicted using PONDR-Fit software (http://original.disprot.org/pondr-fit.php) using sequences for each sortilin variant. Scores over 0.5 are predicted to be intrinsically disordered. (**d**) NDM adipocytes were transfected with Sort_T for 48 h. Conditioned media was collected from NDM, T2DM, or NDM over-expressing Sort_T, methanol extracted and analyzed via Western blot using the Sort_T specific antibody. Ponceau-S staining was used to visualize equal loading of lysates in the lanes. (**e**) Using the protein folding server I-TASSER (https://zhanggroup.org/I-TASSER/) and Schrodinger, models of Sort_FL, Sort_T and Glut4 were generated and equilibrated. Visualization and analysis were carried out using VMD 1.9.3. Characterization of protein–protein interactions between sortilin variants and Glut4 was carried out using ClusPro. Sort_FL was embedded into the membrane at TM domain (Shown as brown bars). Glut4 binding region (G4BR) is shown in blue. Arg (R292, in red) is on end of exon 17 and serves as an orientation point. The additional amino acids on Sort_T are shown in green. (**f**) Coimmunoprecipitation assay was performed by using Glut4 antibody for immunoprecipitation. Western blot analysis was then performed on the immunoprecipitated (IP) samples using antibodies against sortilin (against the N-terminal domain recognizing both variants) or Glut4 as indicated in the figure. Graphs show densitometric analysis of individual sortilin bands normalized to Glut4 (n = 3). Simultaneously, the input lysate was analyzed via Western blot using antibodies against sortilin or Glut4 (n = 3). Statistical analysis was performed via two-tailed *t*-test, ns = not significant.

Available structures of sortilin in the literature and the Protein Data Bank only consist of the VPS10p luminal domain. With the improvement of molecular modeling, especially de novo prediction, we sought to model the complete conformation of sortilin splice variants (Figure 2a) using AlphaFold2 (https://alphafold.ebi.ac.uk/), an artificial intelligence (AI) software by DeepMind [27]. Following 3D structure prediction, the lowest energy folding compositions for Sort_T and Sort_FL were equilibrated for 100 ns using molecular dynamics (MD) performed with NAMD 2.12 [28] using the CHARMM36m force field [29]. Root mean square deviation (RMSD) for sortilin splice variants (Figure 2b) indicates stability of protein conformations over time [30,31]. 

Since insertion of exon 17b occurs between the disordered 10CC motif [32] and the ordered transmembrane domain, we evaluated whether the sortilin exon 17b insertion introduced ordered or amplified the already intrinsically disordered 10CC region. Intrinsically disordered regions (IDRs) have increased solvent exposure due to reduced or absent secondary and tertiary structure. This open conformation may account for more post-translational modifications and potential binding partners, but may affect the overall protein’s function if the domain is necessary for protein–ligand affinity [33]. Using the meta predictor PONDR-Fit (http://original.disprot.org/pondr-fit.php), protein sequences for Sort_FL and Sort_T were analyzed. PONDR-Fit calculates an average disorder score and standard error per residue and thus predicts disorder indicated by scores over 0.5 [33,34]. The results (Figure 2c) show that Sort_FL contains the ordered structure of the transmembrane region (blue) and the intrinsic disordered cytoplasmic domain (ID score over 0.5). The results also demonstrate that the 10 amino acids of Sort_T (marked in red) contribute to the 10CC intrinsic disordered region. 

### 2.6. Sort_T Is Secreted from Adipocytes

Since Sort_T lacks the transmembrane domain, we evaluated whether sortilin is present extracellularly in adipocytes. NDM adipocytes were transfected with Sort_T for 48 h. Conditioned media were collected from NDM, T2DM, or NDM over-expressing Sort_T, methanol extracted and analyzed via Western blot using the Sort_T specific antibody. The results (Figure 2d) show increased Sort_T in media from T2DM and in NDM over-expressing Sort_T compared to NDM adipocytes.

### 2.7. Computational Modeling Predicts Sort_T Maintains Ability to Bind Glut4

The lowest energy conformation structures for Sort_FL, Sort_T, and Glut4 were generated as described above. Previous studies indicate sortilin’s VPS10p domain binds the first loop of Glut4’s luminal domain [15]; thus, computational modeling was used to dock each sortilin splice variant to the first luminal loop of Glut4 using the ClusPro [35] server (https://cluspro.org/). The results (Figure 2e) predict similar Glut4 interaction for both Sort_T and Sort_FL at a site distant from the well-studied inner propeller binding pocket indicated by the Arg-292. The similar interaction conformations suggest the retained binding ability of Sort_T to Glut4. 

### 2.8. Glut4 Binds to Sort_FL and Sort_T

To verify binding of Sort_T to Glut4, a co-immunoprecipitation assay was performed in NDM and T2DM adipocytes. Glut4 antibody was used to immunoprecipitate associated proteins and a Western blot was performed. Antibody targeting sortilin’s N-terminal domain was used to visualize both sortilin variants. The results (Figure 2f) show Glut4 binds both Sort_FL and Sort_T splice variants in NDM and T2DM adipocytes.

### 2.9. Effects of Sortilin Splice Variants on Insulin Stimulated Glucose Uptake

**Figure 3:**
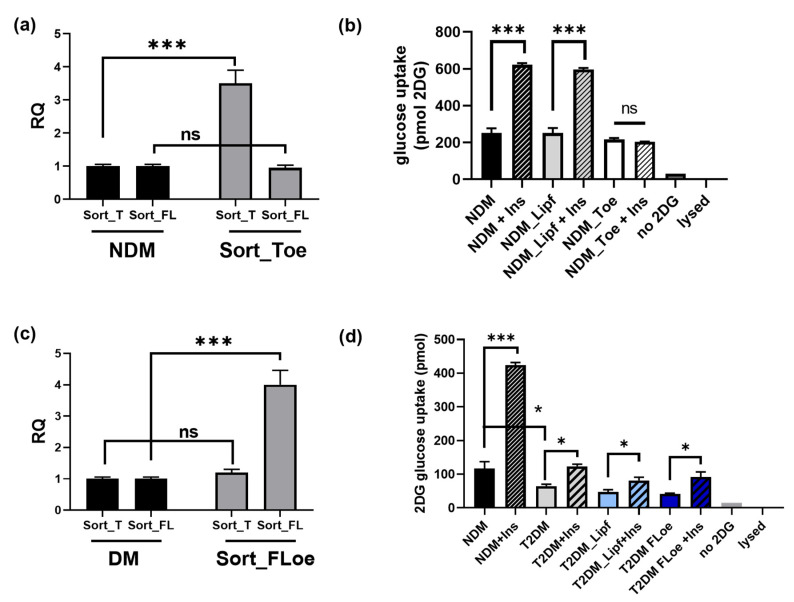
Sort_T overexpression decreases insulin stimulated glucose uptake. (**a**) Sort_T was transfected into NDM adipocytes, and RNA was harvested to confirm overexpression of Sort_T by qPCR analysis. *** *p* < 0.001, ns = not significant. (**b**) Simultaneously Sort_T was overexpressed in NDM adipocytes and glucose uptake assay was performed and uptake of 2 deoxyglucose-6-phosphate: (2DG) was measured; lipofectamine mock transfection, no DG and lysed cells were used as controls (n = 5). Statistical analysis was performed by multiple *t*-tests between basal and insulin treated samples; *** *p* < 0.001, ns = not significant. (**c**) Sort_FL was over-expressed in T2DM adipocytes, and RNA was harvested to confirm Sort_FL overexpression via qPCR analysis. Statistical analysis was performed by multiple *t*-tests between DM and Sort_FLoe samples; *** *p* < 0.001, ns = not significant. (**d**) Simultaneously, glucose uptake assay was performed on T2DM adipocytes over-expressing Sort_FL as described above (n = 3). Controls included NDM adipocytes, lipofectamine mock transfection, no 2DG, and lysed cells. Statistical analysis was performed via multiple *t*-tests between basal nondiabetic and diabetic adipocytes, and basal and insulin treated samples; * *p* < 0.05, *** *p* < 0.001, ns = not significant.

Next, we evaluated the physiological outcome of increased Sort_T levels in adipocytes. Sort_T was overexpressed in NDM adipocytes, then treated with 100 nM insulin followed by glucose uptake assay. Over-expression of Sort_T was verified via real-time qPCR (Figure 3a). Impaired insulin stimulated glucose uptake was seen in NDM adipocytes overexpressing Sort_T compared to control NDM (Figure 3b). 

To determine if increasing Sort_FL levels in T2DM adipocytes could enhance insulin stimulated glucose uptake, Sort_FL was overexpressed in T2DM adipocytes and glucose uptake assay performed. Over-expression of Sort_T was verified via real-time qPCR (Figure 3c). The results (Figure 3d) demonstrate no change in insulin-stimulated glucose uptake with Sort_FL overexpression in T2DM adipocytes compared to control T2DM. 

### 2.10. GLP1 Decreases Exon 17b Inclusion in Sortilin Pre-mRNA

**Figure 4:**
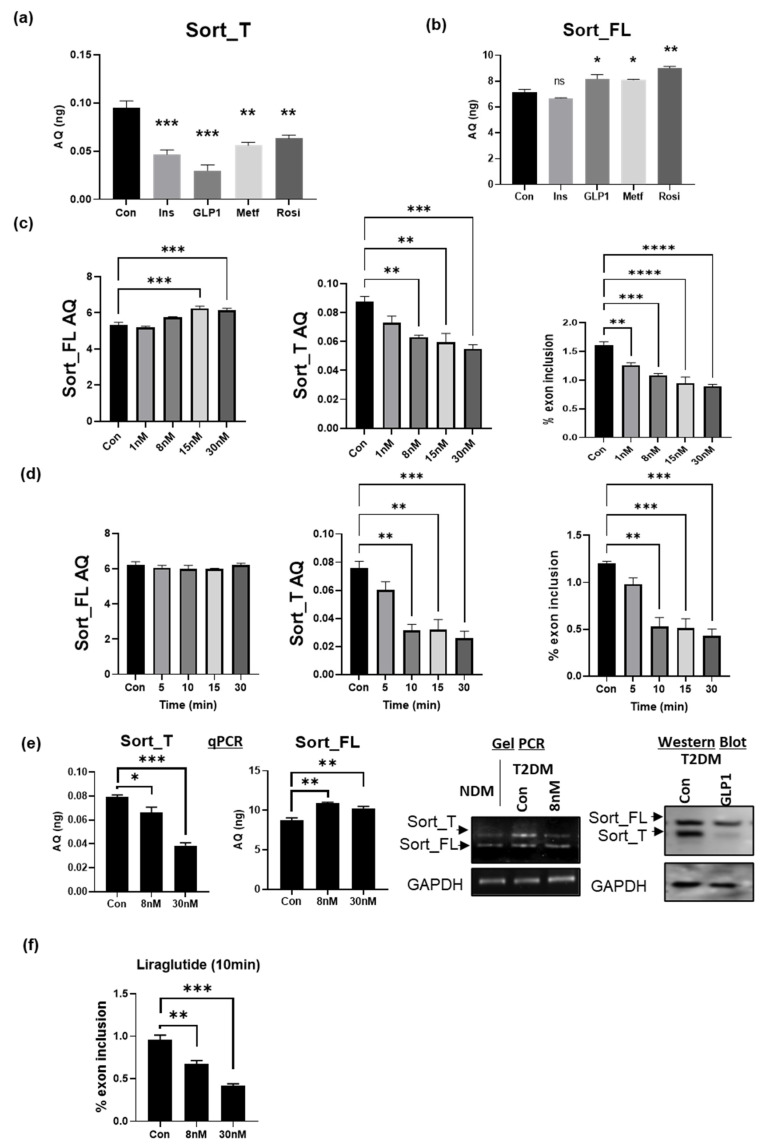
GLP1 reduces Sort_T levels in diabetic adipocytes. T2DM adipocytes and treated with 10 μM metformin (24 h), rosiglitazone (24 h), 100 nM insulin (30 min) or 30 nM GLP1 (30 min). RNA was isolated and qPCR was performed using primers specific to (**a**) Sort_T or (**b**) Sort_FL. To determine absolute quantities (AQ, ng) of each splice variant, a standard curve was included and normalized to GAPDH expression (n = 3). Statistical analysis was performed using one-way ANOVA, comparing each treatment to control. * *p* < 0.05, ** *p* < 0.01, *** *p* < 0.001, ns = not significant. (**c**) T2DM adipocytes treated with increasing doses of GLP1 for 30 min. RNA was isolated and qPCR was performed using primers specific to Sort_T and Sort_FL. A standard curve for each sortilin splice variant was included and absolute quantification (AQ) was determined and normalized to GAPDH levels (n = 3). Percent exon inclusion was determined from absolute quantification of each splice variant using the following equation: $\%exon\;inclusion=\frac{Sort\_T}{Sort\_T+Sort\_FL}*100\%$. Statistical analysis was performed via one-way ANOVA using untreated T2DM adipocytes as control, ** *p* < 0.01, and *** *p* < 0.001, **** *p* < 0.0001. (**d**) T2DM adipocytes were treated with 8 nM GLP1 and RNA was harvested at different time points (n = 3). QPCR was performed using primers specific to Sort_T and Sort_FL. A standard curve for each sortilin splice variant was included and absolute quantification quantified and normalized to GAPDH levels followed by quantification of percent exon inclusion. Statistical analysis was performed via one-way ANOVA using untreated diabetic adipocytes as control, ** *p* < 0.01, and *** *p* < 0.001. (**e**) T2DM adipocytes were treated with 8 nM GLP1 for 30 min. Whole-cell lysates were analyzed via Western blot using antibodies against sortilin (against the N-terminal domain recognizing both variants) or GAPDH (n = 3) or total RNA was used in PCR with primers spanning exon 16 to exon 20 (detecting both variants). Products were separated on 1% agarose gel and visualized using ethidium bromide (n = 3). Separately, real-time qPCR was performed using primers specific to Sort_T and Sort_FL. A standard curve for each sortilin splice variant was included and absolute quantification was determined and normalized to GAPDH levels (n = 3). Statistical analysis was performed via one-way ANOVA using untreated T2DM adipocytes as control, * *p* < 0.5, ** *p* < 0.01, and *** *p* < 0.001. (**f**) T2DM adipocytes were treated with 8 nM or 30 nM Liraglutide, a GLP1 receptor agonist, for 10 min. RNA was isolated and qPCR was performed using primers specific to Sort_T and Sort_FL (n = 3). Percent exon inclusion was determined from absolute quantification of each splice variant using the following equation: $\%exon\;inclusion=\frac{Sort\_T}{Sort\_T+Sort\_FL}*100\%$. Statistical analysis was performed via one-way ANOVA using untreated T2DM adipocytes as control. ** *p* < 0.01, *** *p* < 0.001.

We evaluated therapeutics developed for T2DM management and sought to determine if they may affect alternative splicing of sortilin. To test this, T2DM adipocytes were treated with metformin, rosiglitazone, insulin, or glucagon-like peptide-1 (GLP1). RNA was isolated and qPCR was performed using primers specific to Sort_T or Sort_FL. The results (Figure 4a,b) show all treatments reduced Sort_T expression; however, GLP1 was the most effective at significantly reducing Sort_T levels and increasing Sort_FL in T2DM adipocytes.

Based on these results, we determined the effect of GLP1 on sortilin splice variants by performing a dose and time curve. T2DM adipocytes were treated with increasing doses of GLP1 for 30 min and qPCR was performed. The absolute quantification and percent exon inclusion results (Figure 4c) demonstrate that 8 nM of GLP1 is an optimal dose for reducing exon 17b inclusion into sortilin pre-mRNA in T2DM adipocytes. This was followed by a time curve using 8 nM GLP1 concentration in T2DM adipocytes. Absolute quantification and percent exon inclusion was determined, and the results (Figure 4d) demonstrate a time-dependent decrease in Sort_T expression levels. Next, T2DM adipocytes were treated with GLP1 (8 nM) for 30 min. Cells were simultaneously harvested for Western blot analysis of protein expression of sortilin variants and gel PCR for sortilin splice variant levels. Additionally, RNA from T2DM adipocytes treated with 8 nM or 30 nM GLP1was also evaluated simultaneously for sortilin splice variant expression using real-time qPCR. The results (Figure 4e) show decrease in Sort_T in GLP1 treated T2DM adipocytes.

### 2.11. GLP1R Agonist Liraglutide Decreases Exon 17b Inclusion in Sortilin Pre-mRNA

GLP1 has a short half-life of two minutes and GLP1R agonists have been developed with modifications to increase stability of the peptide. Liraglutide has a 97% sequence homology to GLP1 and has an inclusion of a fatty acid chain and a Lys34Arg substitution, which increases the half-life to 13 h in vivo [36,37,38]. Hence, we evaluated liraglutide’s effects on sortilin’s pre-mRNA alternative splicing. The T2DM adipocytes were treated with 8 nM or 30 nM liraglutide for 10 min. The results (Figure 4f) show a dramatic reduction of Sort_T with a concurrent increase in Sort_FL.

### 2.12. Predicted Splice Factors Regulating Sortilin Alternative Splicing in Diabetic Adipocytes

**Figure 5:**
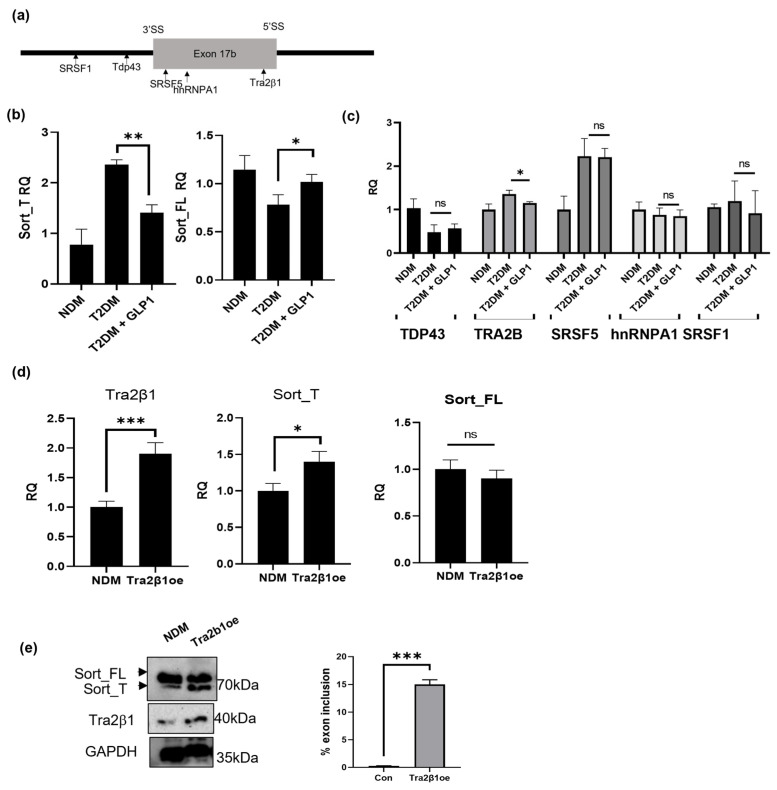
Tra2β1 mediates GLP1-regulated sortilin alternative splicing in diabetic adipocytes. (**a**) Schematic of bioinformatics-predicted splice factor binding on human sortilin exon 17b sequence and upstream intronic sequences. T2DM adipocytes were treated with 8 nM GLP1 for 10 min. RNA was harvested and qPCR was performed (**b**) using primers specific to Sort_T and Sort_FL or primers specific to (**c**) splice factors TDP43, TRA2B, SRSF5, hnRNAA1, and SRSF1. Results were normalized to GAPDH, relative quantification (RQ) was calculated by setting NDM as reference (n = 3). Statistical analysis was performed via two-tailed *t*-test between T2DM adipocytes and T2DM GLP1-treated sample. * *p* < 0.05, ** *p* < 0.01 and ns = not significant. (**d**) NDM adipocytes were transfected with Tra2β1 plasmid (Tra2β1oe) or lipofectamine mock transfection for 48 h. RNA was harvested, and qPCR performed using primers specific to Tra2β1, Sort_FL and Sort_T. Results were normalized to GAPDH, relative quantification (RQ) was calculated by setting NDM as reference (n = 3). Statistical analysis was performed via two-tailed Student’s *t*-test. *** *p* < 0.001, * *p* < 0.05, and ns = not significant. (**e**) Cell lysates were harvested from NDM adipocytes overexpressing Tra2β1 (Tra2β1oe) and mock transfected control cells (NDM). Western blot was performed and immunoblotted using antibodies indicated (n = 3). Graph represents percent exon inclusion from densitometric analysis of blot using the equation: $\%exon\;inclusion=\frac{Sort\_T}{Sort\_T+Sort\_FL}*100\%$. Statistical analysis was performed via two-tailed Student’s *t*-test, *** *p* < 0.001.

Splice factors are trans-acting proteins that are components of the spliceosome. The binding of splice factors to their conserved cis sequences along the pre-mRNA regulates the inclusion or exclusion of the exon by the spliceosome during pre-mRNA splicing. The dynamic interaction of splice factors regulates the pattern of alternative splicing to produce different mRNAs from a single gene. To predict the splice factors involved in sortilin’s alternative splicing, sortilin exon 17b (99bp) and 200, base pairs upstream and downstream (totaling 499 nucleotides), were computationally analyzed using ESEfinder [39] and SFmap [40]. The analysis revealed strong consensus sequences for splice factors hnRNPA1, SRSF5 (aka SRp40), TRA2B (aka Tra2β1), TDP43, and SRSF1 (aka SF2/ASF) (schematic in Figure 5a). 

Since GLP1 treatment decreased the expression of Sort_T, we sought to determine if the expression levels of any of the identified splice factors were affected by GLP1 treatment in T2DM adipocytes in concurrence with the changes in Sort_T expression. T2DM adipocytes were treated with 8 nM GLP1 for 30 min. Real-time qPCR was performed, and the results show increased levels of Sort_T in T2DM adipocytes and a decrease in Sort_T levels in response to GLP1 treatment (Figure 5b). Samples were further analyzed via qPCR for expression levels of hnRNPA1, SRSF5, TRA2B, TDP43, and SRSF1 to identify the splice factor whose expression levels correlated with Sort_T expression levels in GLP1-treated T2DM adipocytes. The results (Figure 5c) demonstrate that TRA2B is increased in T2DM adipocytes and treatment with GLP1 decreases its levels. While the levels of all these splice factors differed between NDM and T2DM adipocytes, the results indicate TRA2B (hereafter referred to as Tra2β1 protein) as the splice factor regulated by GLP1 treatment whose expression pattern changed in concurrence with Sort_T expression levels. 

### 2.13. Tra2β1 Regulates Sortilin Alternative Splicing in Diabetic Adipocytes

Next, we evaluated if Tra2β1 directly increases expression of Sort_T. For Tra2β1 overexpression, NDM adipocytes were transfected with Tra2β1 plasmid for 48 h. The QPCR (Figure 5d) and Western blot analysis (Figure 5e) results demonstrate that Tra2β1 overexpression significantly promotes percent sortilin exon 17b inclusion and an increase in Sort_T protein expression.

### 2.14. GLP1 Decreases Inclusion of Sortilin Exon 17b in a Splicing Minigene

**Figure 6:**
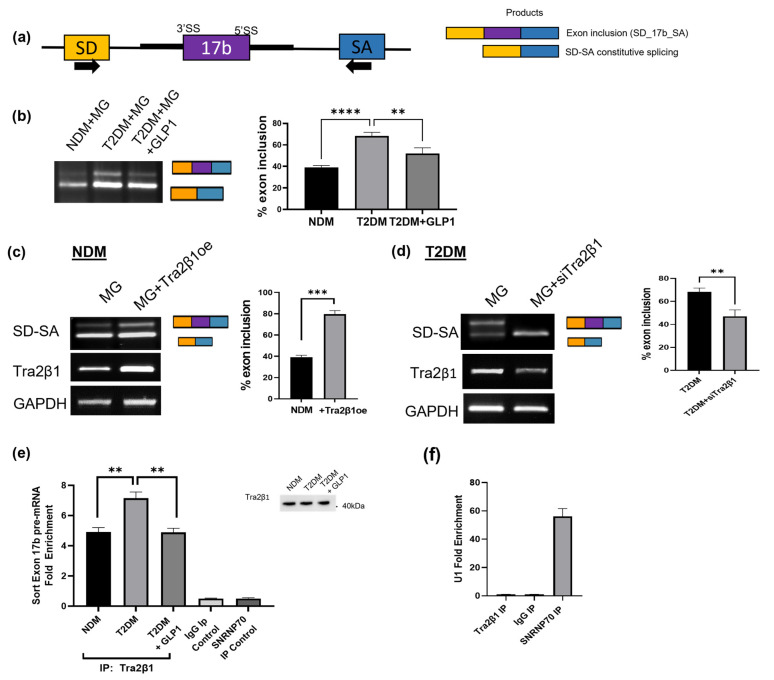
Human sortilin splicing minigene exon 17b inclusion is regulated by GLP1 and Tra2β1 (**a**) Schematic representing the sortilin splicing minigene (MG) with sortilin exon 17b (99 bp) and 200 bp of its flanking 3′ and 5′ introns cloned between exons SD (splice donor) and SA (splice acceptor) on the pSPL3 heterologous splicing vector. The arrows indicate primer positions to generate product with exon 17b inclusion (SD spliced to exon 17b spliced to exon SA) or exon 17b exclusion (constitutive splicing of SD to SA). (**b**) MG was transfected into NDM and T2DM adipocytes followed by GLP1 treatment (8 nM, 10 min) in T2DM (n = 3). RNA was isolated, and PCR was performed using primers on exons SD and SA to yield two products (n = 3). Densitometric analysis was performed, and percent exon inclusion was calculated using the following equation: $\%exon\;inclusion=\frac{SD\_17b\_SA}{SD\_17b\_SA+SD\_SA}*100\%$. Statistical analysis was performed using one-way ANOVA. ** *p* < 0.01 and **** *p* < 0.0001. (**c**) NDM adipocytes were transfected with Tra2β1 plasmid (Tra2β1oe) for 48 h followed by MG transfected for 24 h, RNA was isolated, and PCR was performed using primers on exons SD and SA to yield two products (n = 3). Densitometric analysis was performed, percent exon inclusion was calculated, and statistical analysis was performed using two-tailed Student’s *t*-test, *** *p* < 0.001. (**d**) T2DM adipocytes were transfected with Tra2β1 siRNA (siTra2β1) for 48 h followed by MG transfected for 24 h, RNA was isolated, and PCR was performed using primers on exons SD and SA to yield two products (n = 3). Densitometric analysis was performed, percent exon inclusion was calculated, and statistical analysis was performed using two-tailed Student’s *t*-test, ** *p* < 0.01. (**e**) T2DM adipocytes were treated with 8 nM GLP1 for 10 min. RIP assay was performed by immunoprecipitating with Tra2β1 antibody, IgG antibody as a negative control, and SNRNP70 antibody as a positive control. RNA was eluted from antibodies and qPCR was performed using primers specific for sortilin exon 17b and Western blot was performed on input (shown in inset) using Tra2β1 antibody. ** *p* < 0.01. (**f**) U1, a spliceosome snRNP with known binding to SNRNP70 protein was analyzed by qPCR as a positive control. Analysis to calculate fold enrichment was performed using Ct values and Excel template provided by SigmaAldrich (St. Louis, MO, USA). Statistical Analysis was performed using one-way ANOVA, comparing NDM vs. T2DM, and T2DM vs. T2DM + GLP1.

The heterologous splicing minigene is a tool used to study alternative splicing without the influence of other endogenous factors. Hence, to evaluate if Tra2β1 could regulate the inclusion of sortilin’s exon 17b directly, a sortilin exon 17b minigene was cloned. The pSPL3 splicing vector has two constitutive splicing exons: splice donor (SD) and splice acceptor (SA) exons, with a multiple cloning sites between them. Sortilin exon 17b and 200 bp upstream and downstream (499 bp) were cloned into the multicloning site of the pSPL3 vector and the resulting sortilin splicing minigene (MG) was verified via sequencing. Primer positions (Figure 6a) are depicted by arrows on SD and SA exons. Upon splicing, two products are obtained: a constitutive splicing of SD–SA and a product of 99 base pairs, larger due to the inclusion of sortilin exon 17b between SD and SA exons. The constitutive splicing of SD to SA provides an in-sample control for comparison with exon 17b inclusion.

NDM and T2DM adipocytes were transfected with MG for 24 h and T2DM+MG were treated with 8 nM GLP1 for 10 min. RNA was isolated and PCR was performed using SD and SA primers. The results show an increase in exon 17b inclusion in T2DM adipocytes compared to nondiabetic controls and a decrease in exon 17b inclusion with GLP1 treatment (Figure 6b).

### 2.15. Tra2β1 Increases Inclusion of Sortilin Exon 17b in a Splicing Minigene

To determine the role of Tra2β1 on sortilin exon 17b inclusion, NDM adipocytes were transfected with Tra2β1 plasmid for 48 h, followed by MG transfection for 24 h. PCR was performed using SD and SA primers. The results show an increase in exon 17b inclusion in the MG splicing with Tra2β1 overexpression compared to the lipofectamine control (Figure 6c).

In separate experiments, Tra2β1 was depleted using siRNA in T2DM adipocytes for 48 h followed by MG transfection for 24 h. RNA was isolated and PCR was performed. The results show a decrease in sortilin exon 17b inclusion in the MG splicing with reduced Tra2β1 levels (Figure 6d).

### 2.16. RIP Assay Shows Decreased Tra2β1 Binding to Sortilin Pre-mRNA with GLP1 Treatment

To evaluate Tra2β1 binding to exon 17b on sortilin pre-mRNA, an RNA immunoprecipitation assay (RIP) was performed. NDM and T2DM adipocytes were treated with 8 nM GLP1 for 10 min, then harvested. RIP assay was performed using the Tra2β1 antibody to immunoprecipitate and pull down associated sortilin pre-mRNA. An SNRP70 antibody was used as a positive control and an IgG antibody served as a negative control. A portion of input was removed to confirm equal Tra2β1 protein immunoprecipitation (inset) and RNA was eluted. QPCR was performed using primers on sortilin exon 17b and fold enrichment was calculated (Figure 6e). Fold enrichment for U1 binding (binds SNRNP70 protein) was calculated (Figure 6f) as a positive control. The results show that T2DM adipocytes have increased Tra2β1 binding to sortilin pre-mRNA, which is reduced with GLP1 treatment.

### 2.17. Morpholino Antisense Oligonucleotides Decrease Exon 17b Inclusion and Improve Glucose Uptake in Diabetic Adipocytes

**Figure 7:**
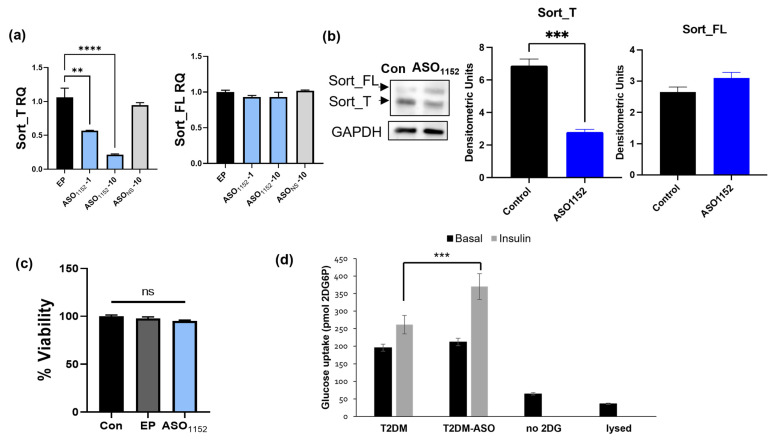
Morpholino antisense oligonucleotides mask Tra2β1 binding site on sortilin pre-mRNA. A Morpholino antisense oligonucleotide was developed to target the Tra2β1 consensus sequence and named ASO_1152_. (**a**) T2DM adipocytes were treated with ASO_1152_ at 1 μM and 10 μM, mock Endo-Porter transfection (EP), and nonspecific antisense oligo control (ASO_ns_) at 10 μM for 48 h. RNA was isolated and qPCR performed using primers specific to Sort_FL and Sort_T and normalized to GAPDH levels and Endo-Porter mock transfection control (n = 3). ** *p* < 0.01, **** *p* < 0.0001. (**b**) Simultaneously, Western blot analysis was performed using antibodies to N-terminal sortilin (detects both Sort_FL and Sort_T simultaneously) and GAPDH (n = 3). Statistical analysis was performed using one-way ANOVA. *** *p* < 0.001, and ns = not significant. (**c**) Acridine orange (AO) and propidium iodide (PI) viability assay was performed on untreated T2DM adipocytes, Endo-Porter mock transfection control, and morpholino ASO_1152_ treated T2DM adipocytes. Stained cells were analyzed in a cellometer (n = 5). Statistical analysis was performed using one-way ANOVA. ns = not significant. (**d**) T2DM adipocytes were treated with ASO_1152_. Insulin-stimulated glucose uptake assay was performed and uptake of 2 deoxyglucose-6-phosphate: (2DG6P) was measured; no DG and lysed cells were used as controls (n = 5). Statistical analysis was performed via multiple *t*-tests between basal and insulin-treated samples; *** *p* < 0.001, ns = not significant.

Morpholinos are antisense oligonucleotides with a modified morpholine backbone, resulting in the ability to influence splicing events without degradation of the pre-mRNA transcript. Morpholino ASOs were designed spanning the Tra2β1 consensus sequence within exon 17b, such that the ASO inhibits Tra2β1 binding to sortilin pre-mRNA. The optimal 25-mer ASO (ASO_1152_) was blasted using an NCBI Nucleotide Blast to confirm uniqueness and minimize off target effects within the human genome. T2DM adipocytes were treated with 1 μM or 10 μM ASO_1152_, or 10 μM of a nonspecific control ASO (ASO_NS_), or Endo-Porter transfection control (EP) for 48 h. The results (Figure 7a) show reduced Sort_T expression with no change in Sort_FL expression levels with ASO. Western blot analysis (Figure 7b) showed decrease in Sort_T protein levels. Apoptosis assay (Figure 7c) demonstrated no toxicity with ASO_1152_. Next, T2DM adipocytes were treated with ASO_1152_, and glucose uptake assay was performed. The results (Figure 7d) show improved insulin-stimulated glucose uptake with the morpholino ASO_1152_ treatment. 

## 3. Discussion

Sortilin is a multiligand receptor and trafficking protein that is constitutively expressed in multiple organs. Previously, Prudencio et al. described a truncated sortilin variant in the brain and showed that in human neuronal cells, the truncated sortilin is a nonfunctional receptor that does not internalize progranulin [22,41]. However, in adipocytes, sortilin functions as a trafficking protein and binds to Glut4 in GSVs. Here, we report that in addition to the constitutively expressed full-length sortilin (Sort_FL), human adipose tissue and adipocytes express the alternatively spliced, truncated sortilin (Sort_T) splice variant. We demonstrate that Sort_T expression is increased in human adipocytes from T2DM individuals. Our data also show that Sort_T is found in the conditioned media of diabetic adipocytes and non-diabetic adipocytes over-expressing Sort_T, which is in concurrence with previous reports [22,41,42,43]. These results suggest that Sort_T is either secreted directly from the trans Golgi network or is included in the GSV budding and held in the cytoplasm. Sort_FL is comprised of a large and highly conserved VPS10p domain (10-propeller structure) at its amino terminus, followed by a single-pass transmembrane domain and a short motif rich C-terminal tail. The 10CC site (comprised of 10 cysteines and 5 disulfide bonds) is directly below the VPS10p domain. The 10CC domain serves as an allosteric site, affecting ligand binding and protein stability [44]. In the alternatively spliced Sort_T variant, exon 17b inserts immediately after the 10CC site, introducing 10 novel amino acids prior to the stop codon, resulting in a truncated protein that lacks the cytosolic tail (schematic shown in Figure 1e). The C-terminal cytosolic tail of Sort_FL binds to retromer complex proteins and regulates movement of GSVs within the trans-Golgi network [18,19,20]. 

Since Sort_T lacks the cytosolic tail, we sought to determine the role of Sort_T in GSVs in adipocytes, which is not yet known. Artificial-intelligence-based structure prediction and molecular modeling indicated that Sort_T retained its ability to bind Glut4, and our coimmunoprecipitation assay results verified this interaction. The overexpression of Sort_T in nondiabetic adipocytes reduced glucose uptake while the over-expression of Sort_FL could not rescue T2DM glucose uptake. Based on these observations, we posit that Sort_T may affect GSV stability and trafficking in adipocytes. In diabetic adipocytes, the lack of sortilin’s C-terminus results in the loss of guiding interactions, including post- translational modifications needed for endosomal sorting. Consequentially, Sort_T and its associated proteins may be transported to the lysosome for degradation, contributing to decreased glucose uptake. We are undertaking experiments such as pulse chase assays and measurements of UPP and lysosomal proteases to evaluate if Sort_T goes through lysosomal degradation with or without Glut4 in T2DM. This is beyond the scope of this manuscript, which is focused on the GLP1-mediated alternative splicing of sortilin. GSV has several components such as IRAP and LRP1, and the interaction of Sort_T with other GSV proteins is unknown and is being investigated by our laboratory. It is also possible that the absence of the cytoplasmic tail of Sort_T may not be solely responsible for aberrant Glut 4 trafficking, as other GSV proteins may compensate for it. It is likely that multiple defects contribute to impaired Glut4 trafficking and the degradation of GSVs in diabetes in adipocytes. 

GLP1 is an incretin hormone normally found in blood at low concentrations (50 pmol/L), whose concentration is rapidly increased (peak levels of 200 pmol/L) following a meal [45]. Basal and postprandial levels of GLP1 are reduced in T2DM despite preservation of GLP1 receptor sensitivities and pathways. GLP1 was identified as a major contributor to the success of bariatric surgery in alleviating insulin resistance [46,47], and the GLP1 response is suggested as a predictor of success for remediation of T2DM via bariatric surgery [48,49,50,51,52]. GLP1 receptor agonists (GLP1Rs), GLP1 analogs, and DPP4 inhibitors behave similarly by preserving or mimicking GLP1 action. This family of T2DM medications shows cardio-protectivity, aids in weight loss via delayed gastric emptying and satiety, and has rare instances of hypoglycemia. 3T3L1 adipocytes treated with GLP1 demonstrate increased adipogenesis, resulting in advantages such as increased levels of Glut4, insulin receptor substrate-1 (IRS1), and insulin receptor β (IRβ) [53,54,55]. Contrasting results are seen in human adipocytes differentiated from human adipose stem cells (hASCs). Human adipocytes treated with GLP1 show a reduction of adipogenesis via a decreased expression of PPAR-γ, CEBP-α, and GSK-3 [56,57,58]. 

Alternative splicing is a post-transcriptional process resulting in the expansion of the transcriptome and proteome of a cell. This dynamic method is orchestrated by a collection of trans factors such as RNA binding proteins and spliceosome components (about 200 proteins associate/dissociate with the spliceosome) recognizing conserved cis sequences on pre-mRNA. Alternative splicing events are unique to tissues, developmental stages, sex, and disease states. The results of alternative splicing are pervasive, affecting every function of the cell through a multitude of means. Serine-arginine rich splice factors (SR proteins) are splicing enhancers known to act as a tether between pre-mRNA transcripts and the spliceosome complex, thus increasing exon inclusion Studies have implicated SR proteins in 5′ splice site selection, binding U1 snRNP, and aiding sequence recognition for splicing [59,60,61]. Tra2β1 is an SR-like protein, also known as SFRS10 or RA301, and is composed of two arginine-serine-rich (RS) domains with an RNA recognition motif (RRM) in between. The TRA2B gene was initially discovered in Drosophila and was named transformer 2β homolog and later renamed SFRS10 with a new SR splice factor nomenclature introduced by Manley and Krainer in 2010 [62]. Tra2β1/SFRS10 is not to be confused with SR splicing factor SRSF10 (also known as TASR1/SRp38/SRrp40). Increased Tra2β1 is associated with circulatory thickening and lesions [63] and is increased with inflammation [64] and nerve injury [65], while Tra2β1 is decreased in liver and muscle where it regulates the splicing of LPIN1 in obese humans and mice [66]. In adipocytes, Tra2β1 affects adipogenesis and enhances lipolysis [67]. Several genes are alternatively spliced in insulin resistance and T2DM [68,69,70]; notably, the insulin receptor gene itself is alternatively spliced and regulated by its own hormone ligand, insulin [71,72]. The data in Figure 5b,c show that the splice factor, TDP43, is reduced in T2DM, concurrent with increased Sort_T levels. Our unpublished data show that over-expressing TDP43 reduces Sort_T levels in adipocytes, which is in concurrence with previous reports on neurons [22,41]. However, GLP1 treatment did not affect TDP43 levels but only affected Tra2β1 levels as shown in Figure 5c. Hence, we pursued the evaluation of Tra2β1 as the splice factor regulated by GLP1 treatment in T2DM adipocytes, as we sought to evaluate the mechanism of the GLP1-mediated alternative splicing of sortilin in adipocytes.

Here, we demonstrate that the incretin hormone, GLP1, regulates the alternative splicing of sortilin in human adipocytes. Hormones such as insulin and GLP1 and insulin-sensitizers such as metformin and rosiglitazone affect kinases and phosphatases that phosphorylate/dephosphorylate trans-factors in the spliceosome, thereby changing their interaction (promote or inhibit) with the cis-element (consensus sequences) on the pre-mRNA. These often affect common signaling pathways such as PKC, PKA, PI3K/AKT that cross-talk to mediate the biological outcome. GLP1 mediates its effects via a G-protein-coupled receptor and signals through the PI3K/AKT, PKA/cAMP, and PKC pathways [73,74]. Previous studies have indicated GLP1 inducing rapid changes (<15 min) in the ERK/MEK pathway in adipocytes compared to slower changes in the PI3K/AKT signaling (2 h) [75]. GLP1 receptor (GLP1R) expression is higher in visceral adipose tissue compared to subcutaneous adipose tissue [76]. GLP1-GLP1R signaling in adipocytes is also shown to be mediated via the PKA and MAPK pathways in adipocytes [75,77]. Suppression of GLP1R expression reduces proliferation and differentiation while inducing apoptosis of pre-adipocytes [75]. GLP1 has also been shown to mediate its effects through the IL6 pathway in human adipocytes [78]. We are currently evaluating whether GLP1 signaling may also affect kinases that phosphorylate splicing trans-factors and whether it may potentially affect alternative splicing events through the signaling cascades in adipocytes.

A widely used method to alter alternative splicing is the use of antisense oligonucleotides (ASOs) to mask an enhancer or silencer binding site, resulting in exon skipping. Morpholinos ASOs are comprised of a morpholine backbone to protect the ASO from RNAse H recognition and cleavage [79,80]. An outcome of modified backbones is the ability to modulate exon inclusion/exclusion without the degradation of the whole pre-mRNA transcript in the nucleus [81,82]. Our results demonstrate that morpholino ASO, targeted to mask the Tra2β1 binding site on exon 17b, reduces Sort_T expression and improves glucose uptake. This may be developed into a new therapeutic option for the management of T2DM.

The in vivo environment in adipose tissue in individuals with type 2 diabetes is, however, varied due to the polygenic nature of the disease. Hence, the limitation of this study is that a direct effect of GLP1, modulating sortilin splicing and culminating in improved glucose uptake in patients with type 2 diabetes, is yet to be demonstrated in vivo. Additionally, Sort_T may influence the formation or trafficking of GSV, which may be affected when sortilin splice variant levels are changed. The signaling pathway of GLP1 as well as the levels and mechanisms of association with its receptor (GLP1R) or signaling via other receptors, to regulate alternative splicing of sortilin, remains to be established in human adipocytes. In summary, we have identified sortilin alternative splicing as an important regulatory process in human adipocytes, and our data indicate that high levels of truncated sortilin substantially contribute to the manifestation of comorbidities associated with type 2 diabetes. Using robust tools and assays, we have demonstrated that GLP1 regulates the alternative splicing of sortilin. The study has established that truncated human sortilin has physiological consequences in glucose uptake in adipose tissue and may be a viable target in the management of type 2 diabetes.

## 4. Materials and Methods

### 4.1. Cell Culture

Human-visceral-adipose-derived stem cells (HASCs) and media were purchased from Zenbio^TM^ (Durham, NC, USA) and passaged as pre-confluent cultures in pre-adipocytes media at 37 °C and 5% CO_2_. Normal (HbA1c < 5.7%), and diabetic hASCs (HbA1c > 7%) were from pooled donors and were BMI matched. On day 2, media were changed to differentiation media, and cells were harvested on day 6 (4 days of differentiation) as differentiated adipocytes for experiments. For mature adipocytes, cells were kept in differentiation media for 6 days and changed to adipocyte media for 48 h before harvesting on day 10.

### 4.2. Human Adipose Tissue

Visceral adipose tissue from male/female nondiabetic/diabetic donors (de-identified, discarded samples from elective surgeries, BMI-matched, diabetic HbA1c > 6.5, nondiabetic HbA1c < 6.5, IRB #Pro00020295: non-human subject research (NHSR)) was homogenized in a bead homogenizer. Whole-cell lysates were harvested or RNA was isolated using Trizol (ThermoFisher Scientific, Waltham, MA, USA) per manufacturer’s instructions. 

### 4.3. Plasmid Transfections

Human sortilin plasmids for Sort_FL and Sort_T were designed by our lab and purchased from VectorBuilder (Chicago, IL, USA). HASCs were grown to confluency and differentiated for 2 days in differentiation media, 1 μg of plasmid per 1 mL of media, or lipofectamine mock transfection control was performed following manufacturer’s instructions (Lipofectamine 3000, ThermoFisher #L3000015) for 48 h in differentiation media, total 4 days of differentiation.

### 4.4. Polymerase Chain Reaction

RNA was isolated using Trizol^TM^ (ThermoFisher # 15596026) and cDNA was synthesized using 1 µg RNA (260/230 > 1.8 and 260/290 > 1.8) using iScript^TM^ (Bio-Rad #1708891). Target amplification was performed with 1 µL cDNA was and JumpStart REDTaq ReadyMix Reaction Mix (Sigma-Aldrich #P0982, St. Louis, MO, USA). Products were separated on a 1% agarose gel, visualized using ethidium bromide, and imaged on the ProteinSimple FluorChem^TM^. Densitometric analysis was performed using AlphaView Software (Version 3.3.0).

Human primers used included hSortilin exon 16 S to hSortilin exon 20 AS to see both sortilin splice variants simultaneously. For qPCR analysis and absolute quantification, human primers used included hSortilin exon 17b S and exon 17b AS to detect Sort_T or hSortilin, exon 17 and exon 17/18 AS to detect Sort_FL. See Table 1 for all human primer sequences. SYBR Green qPCR was performed on the ViiA 7 (ABI) and primer concentrations were optimized for a single melt curve and consistent amplification. Plate set-up included a standard series, no template control, and no reverse transcriptase control. A standard curve was generated and used to calculate absolute quantities of target expression using primers as per experiment. Samples were normalized to GAPDH.

Relative quotient (RQ) was determined using the comparative method (∆∆CT). Primers sequences are shown in Table 1 below. Primers were either purchased from Origene (Rockville, MD, USA) or designed using the primer-designing tool Primer BLAST (NCBI) including verification of primer specificity, followed by synthesis by Eurofins Genomics (Louisville, KY, USA). Next, the optimal primer concentration for forward and reverse primers was determined from a range of 50–900 nM. The final concentration was selected to ensure efficiency and specificity for its target, based on the dissociation curve that showed a single sharp peak, indicating that the primers amplify one specific target.

### 4.5. Western Blot Analysis

Cell lysates were harvested using lysis buffer (Cell Signaling 9803S, Danvers, MA, USA) + 10% protease/phosphatase inhibitor (Pierce A32957, A32953 purchased from ThermoFisher Scientific, Waltham, MA, USA). Conditioned media were collected from cells, mixed with 4 times the volume of methanol, and vortexed well. A volume of chloroform was added and vortexed followed by centrifugation of fat at 9000× *g* for 5 min. The interface layer was carefully separated, mixed with 3 times the volume of methanol, followed by centrifugation of fat at 9000× *g* for 10 min at 4 °C. The supernatant was decanted and the pellet was reconstituted in lysis buffer. Lysates were kept on ice or stored in the freezer for an hour, then sonicated briefly. Cell lysates (80 µg) were separated on a 7.5% SDS-PAGE gel, wet-transferred to nitrocellulose membranes, blocked with 5% nonfat dried milk in Tris-Buffered Saline with 0.05% Tween 20 (TBST). Membranes were probed with Sortilin N- terminal antibody to visualize both splice variants (R&D Systems MAB3154; RRID: AB_2192601, Minneapolis, MN, USA), Sortilin C- terminal antibody to visualize full length sortilin (Abcam #ab16640; RRID: AB_2192606, Cambridge, UK), Glut4 (Cell Signaling #2213; RRID: AB_823508, Danvers, MA, USA), and GAPDH (Santa Cruz #sc25778; RRID: AB_10167668, Dallas TX, USA). The Sort_T antibody was custom made by Pacific Immunology (Ramona, CA, USA) against a synthetic peptide, CSPEKQDSHPQGHSLS, in New Zealand white rabbits. The serum was passed through a peptide affinity column to purify the monoclonal antibody. The specificity for Sort_T antibody was determined using ELISA with pre-immune serum as a control. It was verified via a competition assay with the peptide epitope followed by Western blot analysis.

Secondary HRP antibodies for rabbit (Biorad #5196-2504, Hercules, CA, USA) and mouse (Biorad #0300-0108P, Hercules, CA, USA) were used with chemiluminesence reagent (Pierce #32109 purchased from ThermoFisher Scientific, Waltham, MA, USA) for detection. Images were digitally captured using ProteinSimple FluorChem^TM^ and densitometric analysis was performed using AlphaView Software (version 3.3.0).

### 4.6. Coimmunoprecipitation Assay

Human nondiabetic and diabetic pre-adipocytes were grown to confluency and differentiated for 4 days in differentiation media. Cells were harvested and 200 µg in 200 μL volume of lysate was used for coimmunoprecipitation assay. Lysates were rocked with Protein A/G Agarose (Santa Cruz sc2003, Dallas, TX, USA) for 30 min at 4 °C to clear nonspecific binding then centrifuged at 2000 rpm for 1 min to remove protein beads. Immunoprecipitation was performed using 2 μg Glut4-agarose antibody (Santa Cruz sc53566 AC, Dallas TX, USA) or IgG (Santa Cruz sc-51993, Dallas, TX, USA) as control and rocked overnight at 4 °C. Lysate was then centrifuged, and pellet was washed with PBS and resuspended in Laemmli buffer. Western blot analysis was performed as described above.

### 4.7. Glucose Uptake Assay

Human nondiabetic and diabetic pre-adipocytes were grown to confluency in a 96-well plate and differentiated for 2 days in differentiation media; 1μg plasmid per 1 mL of media for Sort_T, Sort_FL or lipofectamine control was overexpressed for 48 h in differentiation media. Glucose uptake was performed (ProMega #TM467, Madison, WI, USA) by measuring luciferase luminescence. Briefly, cells were serum-starved in Krebs-Ringer Phosphate HEPES buffer (KRPH) for an hour before insulin treatment (100 nM, 10 min). Cells were washed and treated with 1 mM of 2-deoxyglucose 6-phosphate (2DG6P) for 10 min. Cells were washed again in KRPH buffer and lysed. A standard curve was generated, and luminescence was measured on a Biotek Synergy Mx microplate reader (Agilent Technologies, Santa Clara, CA, USA) using a 1 s integration time and 2DG6P quantification of samples were calculated.

### 4.8. Computational Modeling

Sequences for full-length human Glut4 and Sort_FL were obtained from UniProt^232^ and Sort_T alternatively spliced variant sequence was derived via translation of Sort_T nucleotide sequence and alignment with Sort_FL. Sequences were submitted to Alphafold 2.2.0 for protein folding and structure prediction. Folded sortilin variants and Glut4 proteins were minimized in Schrodinger protein preparation wizard (2019-1: Maestro, Schrödinger, LLC, New York, NY, USA). Molecular dynamics simulations were performed using NAMD 2.12 and CHARMM36m force field at a 100 ns time scale. The system was prepared using the CHARMM-GUI solution builder, with a salt concentration of 150 mM NaCl. Visualization and analysis were carried out using Visual Molecular Dynamics (VMD 1.9.3). The system was equilibrated for 100 ns, restraining the Cα atoms of the protein (1.0 kcal/mol/A2) to allow for solvation. Root mean square deviations (RMSD) using backbone Cα atoms were calculated using VMD RMSD trajectory tool (VMD 1.9.3)for Sort_FL, and Sort_T. Protein–protein docking was performed using the last frame from the equilibrated structures of Sort_T, Sort_FL, and Glut4 on the ClusPro server, targeting the first luminal loop of Glut4 interaction to Sort_FL and Sort_T.

### 4.9. Disorder Analysis

Sortilin protein sequences were analyzed using PONDR-Fit (www.disprot.org accessed on May 2019), a multi-residue meta-predictor of intrinsic disorder in protein sequences. PONDR-Fit uses an 8-fold cross-validation method to calculate a disorder score and standard error per residue. The results were exported from the PONDR-Fit server; prediction scores above 0.5 are considered disordered.

### 4.10. Cloning Human Sortilin Exon 17b-Splicing Minigene

The human-sortilin-splicing minigene (MG) was cloned by isolating genomic DNA from human adipose stem cells (hASCs) and amplifying exon 17b ± 200 bp of the upstream and downstream introns using primers with designed SacI and NheI restriction sites. The product and the pSPL3 splicing vector were digested with NheI and SacI restriction enzyme, followed by ligating the insert into the pSPL3 splicing minigene. The orientation of insert was verified via PCR and sequencing of the minigene.

### 4.11. RNA-Immunoprecipitation Assay (RIP)

Imprint RNA Immunoprecipitation kit (Sigma-Aldrich #RIP, St Louis, MO, USA) was purchased and assay was carried out per manufacturer’s instructions. Briefly, human nondiabetic and diabetic adipocytes were differentiated into adipocytes and diabetic adipocytes were treated with GLP1 (8 nM, 10 min); 2 × 10^6^ cells were resuspended in 525 μL RIP Lysis buffer. Cells were harvested then rocked in protein A/G beads for 1 h to clear nonspecific binding. Immunoprecipitation was performed with 2 μg Tra2β1/SFRS10 antibody, SNRNP70 antibody (positive control, Millipore #03-103), or IgG antibody (as a negative control, included in kit). A portion was removed to confirm equal immunoprecipitation via Western blot. RNA was eluted, isolated, and treated with DNase to remove genomic DNA. SYBR Green qPCR was performed using primers specific to sortilin exon 17b and U1 (positive control for SNRNP70). The yield (percent input) and specificity (fold enrichment) were calculated using a provided RIP Excel template from Sigma-Aldrich (St. Louis, MO, USA).

### 4.12. Morpholino Treatment

Human diabetic pre-adipocytes were grown to confluency in a 12-well plate and differentiated for 2 days in differentiation media. Morpholinos were added to fresh differentiation media, to a final concentration of 1 μM or 10 μM, and dish was swirled, then Endo- Porter (Gene Tools, Philomath, OR, USA) 6 μL/mL of media was added and swirled. Cells were incubated for 48 h. Sequences used for the 3′ splice site are 5′-TGTGTGTATGATTGCACAGGACTCC-3′ and for the 5′ splice site, 5′-AGAAGCAGGTAGGTTACACGAAAAC-3′.

### 4.13. Statistical Analysis

Densitometric analysis of Western blots was carried out using AlphaView™ software (Version 3.3.0) from ProteinSimple™. Experiments were independently repeated three to five times for reproducibility. PRISM software (version 10.0.2) (GraphPad) was used for statistical analysis. Two-way analysis of variance (ANOVA) or Student’s *t* test was used in the analysis, indicated by the figure legends (* *p* < 0.05, significant; ** *p* < 0.01, highly significant; *** *p* < 0.001, extremely significant). Each data set passed the Shapiro–Wilk normality test (alpha = 0.05).

## Figures and Tables

**Table 1:** Human Primers with sequence used for PCR analysis and sequencing.

Gene	Species	Forward and Reverse Primer Sequences (5′-3′)
β-Actin	Human	CTCTTCCAGCCTTCCTTCCT AGCACTGTGTTGGCGTACAG
Sortilin exon 16–20	Human	AAGGGCCACGACCTGGAGTT CAATCTCGAGCTATTCCAAGAGGTCCTCATCTG
Sort_T	Human	TTGTCCTAGAATCCAGCTCTGC TCTGGGTGGGAAATAGGGAGT
Sort_FL	Human	TGGGGTAAATCCAGTTCGAG GACTTGGAATTCTGTTTTTCCGGAC
GAPDH	Human	GATCATCAGCAATGCCTCCT TGTGGTCATGAGTCCTTCCA
TRA2B	Human	TGTCTACTCGCAGGCGTCATGT AGACACATCGGCAATGGGACCA
SD-SA	N/A	GTGAACTGCACTGTGACAAGC CACCTGAGGAGTGAATTGGTC

## Data Availability

Full size blots for all figures are uploaded with the paper. No new data were created or analyzed in this study. Data sharing is not applicable to this article.
